# Improving primary health care facility performance in Ghana: efficiency analysis and fiscal space implications

**DOI:** 10.1186/s12913-017-2347-4

**Published:** 2017-06-12

**Authors:** Jacob Novignon, Justice Nonvignon

**Affiliations:** 1 Department of Economics, Kwame Nkrumah University of Science and Technology, Kumasi, Ghana; 2 Department of Health Policy, Planning and Management, School of Public Health, University of Ghana, Legon, Ghana

**Keywords:** Primary health care, Efficiency, Stochastic frontier analysis, Fiscal space for health, Matching decomposition, Ghana

## Abstract

**Background:**

Health centers in Ghana play an important role in health care delivery especially in deprived communities. They usually serve as the first line of service and meet basic health care needs. Unfortunately, these facilities are faced with inadequate resources. While health policy makers seek to increase resources committed to primary healthcare, it is important to understand the nature of inefficiencies that exist in these facilities. Therefore, the objectives of this study are threefold; (i) estimate efficiency among primary health facilities (health centers), (ii) examine the potential fiscal space from improved efficiency and (iii) investigate the efficiency disparities in public and private facilities.

**Methods:**

Data was from the 2015 Access Bottlenecks, Cost and Equity (ABCE) project conducted by the Institute for Health Metrics and Evaluation. The Stochastic Frontier Analysis (SFA) was used to estimate efficiency of health facilities. Efficiency scores were then used to compute potential savings from improved efficiency. Outpatient visits was used as output while number of personnel, hospital beds, expenditure on other capital items and administration were used as inputs. Disparities in efficiency between public and private facilities was estimated using the Nopo matching decomposition procedure.

**Results:**

Average efficiency score across all health centers included in the sample was estimated to be 0.51. Also, average efficiency was estimated to be about 0.65 and 0.50 for private and public facilities, respectively. Significant disparities in efficiency were identified across the various administrative regions. With regards to potential fiscal space, we found that, on average, facilities could save about GH₵11,450.70 (US$7633.80) if efficiency was improved. We also found that fiscal space from efficiency gains varies across rural/urban as well as private/public facilities, if best practices are followed. The matching decomposition showed an efficiency gap of 0.29 between private and public facilities.

**Conclusion:**

There is need for primary health facility managers to improve productivity via effective and efficient resource use. Efforts to improve efficiency should focus on training health workers and improving facility environment alongside effective monitoring and evaluation exercises.

**Electronic supplementary material:**

The online version of this article (doi:10.1186/s12913-017-2347-4) contains supplementary material, which is available to authorized users.

## Background

Primary health care (PHC) is considered to be an important tool in achieving universal health coverage (UHC). Following the Alma Ata declaration in 1978, several countries have embraced the idea of improving PHC to enhance effective health service delivery [1]. In resource-constrained regions like sub-Sahara Africa (SSA), primary health service delivery helps to bridge the health care inequity gap against deprived and vulnerable populations. Effective PHC service delivery also reduces pressure on higher level facilities and hence financial pressure on governments [2].

However, despite its relevance, most primary health facilities are faced with numerous challenges that hinder their effective operations. One such challenge across several countries in SSA is inadequate resources committed to facilities at this level [3]. While recent calls to increase resources committed to these facilities are valid, other public sector priorities compete for these limited resources. It has, therefore, become imperative to create additional fiscal space for the health sector through various means. One of these means is improving efficiency in the operations of facilities and saving resources that can be reinvested into the health sector [4]. Inefficiency means that current facility outputs could be achieved with fewer resources. Improved efficiency shows reduced wastes in the use of already limited resources [5].

Evidence suggests that inefficiency across health facilities is wide spread in developing countries. For instance, Jehu-Appiah et al. [6] provided evidence from district hospitals in Ghana to show that about 56.2% of the 128 hospitals had efficiency scores below average (0.50). The authors also found quasi-government hospitals to be more efficient relative to private and mission hospitals. Similarly, Alhassan et al. [7] analyzed technical efficiency across public and private primary health facilities accredited by the National Health Insurance Authority. About 31% out of the 64 hospitals were estimated to be optimally efficient. There were also more publicly owned facilities that were efficient compared to private and mission hospitals. Interestingly, the study identified higher levels of wastage in urban facilities than rural facilities. Evidence outside Ghana also suggest similar finding. In Burkina Faso, Maschall and Flessa [8] found that less than half of the facilities studied were found to be optimally efficient. Efficiency scores ranged between 1.00 and 0.25 across all facilities. The empirical evidence summarized above point to the fact that there exist significant inefficiencies across health facilities in developing countries and Ghana is no exception. Several other studies support these findings [9–13]

While most of these studies have assessed efficiency at the level of district hospitals, they are not the lowest in the health care cadre in Ghana. Health centers and Community Health Improvement Service (CHIPS) compounds, which are located at the bottom of the health care pyramid, are designed to meet the basic health care needs of the population especially in rural areas. In some cases, district facilities serve as reference facilities for the health centers and CHIPS compounds. In this regard, improving the effectiveness of facilities at the lowest level is key to achieving PHC objectives in Ghana. Unfortunately, inefficiency at this level have been under researched even though these are supposed to serve as first line of treatment in the health care pyramid. Moreover, improving efficiency at this level of the health service delivery system could be an important source of creating fiscal space in the health sector. It is worth noting that potential resource saving from efficiency do not increase the general resource envelop like tax revenue or aid but rather creates space within the existing envelope. It is, therefore, important to understand the nature of inefficiencies that exist in these facilities. The current study contributes to existing studies in three ways; (i) first, it estimates efficiency among primary health facilities (health centers), (ii) secondly, it investigates the efficiency disparities in public and private facilities (iii) finally, it examines the potential fiscal space from improved efficiency.

## Methods

### Data

Data for the study was sourced from the 2011 Access, Bottlenecks, Cost and Equity (ABCE) project conducted by the Institute for Health Metrics and Evaluation (IHME) in collaboration with Ghana Ministry of Health (MOH), Ghana Heath Service (GHS), Ghana UNICEF office and UNICEF. The data collects information from facilities at all levels of the health sector across several countries. Information collected include facility finances and input, facility management, equipment and capacity as well as facility outputs. A total of 87 health centers were included in the final sample. The inclusion of facilities was determined by data availability.

### The stochastic frontier analysis (SFA) model

Following Danquah et al., the starting point of the SFA model is to specify a relationship between a set of health facility inputs that produce an output [14]. This can be specified as

1 $$ {y}_i= f\left({l}_i,{k}_i,{q}_i\right) $$

Where *y* is the output measure (in this case, number of out-patient visit), *l* is labour (total number of clinical and non-clinical staff), *k* is capital (proxied with number of hospital beds and rooms) and *q* is a set of other potential determinants of output (such as age of health facility).

Assuming that some heath facilities do not efficiently employ inputs to produce output, we can capture the actual observable output in the following stochastic frontier production function

2 $$ {y}_i= f\left({l}_i,{k}_i,{q}_i;\beta \right){TE}_i{e}^{v_i} $$

Where β is an estimable parameter, TE captures technical efficiency and measures the deviation of a health facility from the stochastic frontier. *v*_*i*_ is a random error term. Equation (2) can be re-specified in a log-linear form as follows

3 $$ \mathit{\ln}{y}_i=\mathit{\ln} f\left({l}_i,{k}_i,{q}_i;\beta \right)+\mathit{\ln}{TE}_i+\mathit{\ln}{e}^{v_i} $$

Assume *TE*_*i*_ = exp(−*u*_*i*_) we can reformulate Eq. (3) as

4 $$ \mathit{\ln}{y}_i=\mathit{\ln} f\left({l}_i,{k}_i,{q}_i;\beta \right)+{e}_i-{u}_i,\kern1.75em {u}_i>0 $$

As noted earlier, the error term is composed of a sum of normally distributed disturbance (*v*_*i*_) which accounts for measurement and specification error and a one-sided disturbance (*u*_*i*_) which measures inefficiency. Both *v*_*i*_ and *u*_*i*_ are assumed to be independent and identically distributed across observations. An exponential assumption [*u*_*i*_ ~ *ε*(*δ*_*u*_)] proposed by Meensen and VanBroeck [15], was made about the distribution of the inefficiency term [16].

The estimation of the stochastic frontier (SF) requires a functional form of the production function. Two specifications are popular in the literature; the Cobb-Douglas (CD) and Tanslog functional forms. We specify the CD production function in this study as

5 $$ {y}_i=\beta {l}_i^{\alpha}{k}_i^{\pi}{q}_i^{\theta},\kern1em \alpha +\pi +\theta =1 $$

All inputs are as defined in Eq. (1) above. α, π and θ are parameters to be estimated. Equation (5) can be linearized to generate a linear production function as follows;

6 $$ \mathit{\ln}{y}_i=\mathit{\ln}{\beta}_0+\alpha ln{l}_i+\pi {k}_i+\theta {q}_i+{\varepsilon}_i $$

Where *ε*_*i*_ is an error term that can be decomposed into a normal white noise error term and an inefficiency term.

The Translog form of the above production function can be specified as

7 $$ \mathit{\ln}{y}_i=\mathit{\ln}{\beta}_0+\alpha ln{l}_i+\pi {k}_i+\theta {q}_i+0.5\left[{\alpha}_1{ l n}^2{l}_i+{\pi}_1{ l n}^2{k}_i+{\theta}_1{ l n}^2{q}_i\right]+0.5\left[{\alpha}_2\mathit{\ln}{l_i}^{\ast}\mathit{\ln}{k}_i+{\alpha}_3\mathit{\ln}{l_i}^{\ast}\mathit{\ln}{q}_i+{\alpha}_4\mathit{\ln}{q_i}^{\ast}\mathit{\ln}{k}_i\right]+{\varepsilon}_i $$

We estimated both the CD and Translog production functions. To decide on which specification is appropriate, we used the likelihood ratio test. The test was conducted on the null hypothesis that the multiplicative terms in the Translog function are simultaneously equal to zero. Results of the likelihood ratio test (LR Chi2 = 7.26; *p*-value = 0.7011) suggests that the CD function is favorable relative to the Translog function. The lack of statistical significance (high *p*-value) indicates that we fail to reject the null hypothesis. This implies that the CD functional form is sufficient for our analysis. We, however, reported results from the Translog specification as a Additional file 1.

The choice of SFA over the data envelopment analysis (DEA) was motivated by its flexibility to allow control variables aside the direct inputs. Further, while the DEA is the most used in the estimation of health system efficiency among the two models, it is weak in the sense that it is extremely sensitive to the presence of outliers, which define the frontier. Its nonparametric nature also implies that it is unable to address random variations in the data which are then captured as inefficiency. While the SFA addresses these weaknesses, it is also limited in the imposition of some functional form on the production function which, in some cases, become difficult to estimate [17–20]. As discussed earlier, we minimize this weakness in the SFA by statistically deciding which functional form is appropriate for this study.

#### Choice of inputs and outputs

The choice of inputs and outputs was mainly based on availability of data. A search of the literature suggested many variables considered to be standard measures of facility inputs and outputs. Following these, the current study used the following variables in measuring inputs and outputs at the primary health facilities.

*Output variable:* Number of outpatient visits was used as the output variable of interest. This is because most health centers in Ghana only provide outpatient services. Inpatient services are mostly not available at this level of health care.

*Input variable:* The main input variables used in the efficiency estimation include number of personnel, hospital beds and expenditure on other capital items and administration. Other control variables used include rural/urban location, public/private facility type, age of facility, display of fee list and number of rooms available.

### Computing efficiency gain

The potential gains from efficiency was computed by finding the proportion of a facility’s revenues (*R*_*i*_) that could be saved if efficiency was improved. This is presented in Eq. (2) as the proportion of facility revenue that is lost to inefficiency.

8 $$ {rev}_i=\left({eff}_{max}-{eff}_i\right)\times {R}_i $$

where *rev*_*i*_ represents revenue of the *ith* facility that could be gained if inefficiencies were corrected, *eff*_*max*_ is maximum efficiency level (1.00 in this case) and *eff*_*i*_ is actual efficiency score of the *ith* facility, predicted from the SFA specification above.

The potential savings in total facility revenue (*rev*_*i*_) also shows the potential fiscal space for health available for the *ith* facility if efficiency were improved.

### Nopo matching decomposition

Disparities in efficiency between public and private facilities was estimated using the Nopo matching decomposition approach. The Nopo procedure even, though similar to the famous Oaxaca-Blinder approach, is considered to be better for two main reasons. One is that it is fully parametric and in the case of the current study it requires a linear regression model for efficiency. Secondly, it does not restrict comparison to facilities with comparable characteristics, i.e. it ignores the common support problem.

The Nopo decomposition approach uses an algorithm to match a public facility with a similar private facility at the primary level. This implies that the facility type becomes the treatment variable in this decomposition analysis. Four steps were followed to complete the procedure.^1^ These are outlined below;

1. Select one public facility without replacement from the sample
2. Now select all private facilities that have similar characteristics as the public facility selected in step 1
3. Construct a synthetic facility from all the facilities from step 2 whose characteristics are equal to average of all of them and match it to the facility in step 1.
4. Put the observations of both facilities (the synthetic private and the public facility) in their respective new samples of matched facilities
5. Repeat steps 1 through 4 until it exhausts the original public facility sample

Following this characterization, the disparities in efficiency between the matched public and private facilities was then computed. This total gap (Δ) was further disaggregated into four components as described below

Δ_0_ = This is the part of the efficiency gap that cannot be explained by differences in facility characteristics. This is also considered to be the residual part of the decomposition.

Δ_pu_ = This component explains the disparities between public facilities that are matched and those that are unmatched

Δ_pr_ = Similarly, this component shows differences between matched private facilities and unmatched private facilities

Δ_x_ = This component shows that part of the efficiency gap between private and public facilities that can be explained endogenously (differences in covariates).

## Results

### Descriptive statistics

Table 1 presents descriptive statistics of variables (including inputs and outputs) used in the analysis. The averages, standard deviation (Std. Dev.), minimum (Min) and maximum (Max) values were computed and reported. The statistics show that average total annual facility expenditure in 2011 was GH₵94,619.40 (US$63079.60) with minimum and maximum values of GH₵12,709.96 (US$8473.31) and GH₵613,378.40 (US$408,918.93), respectively.

**Table 1:** Descriptive statistics

Variable	Observation	Mean	Std. Dev.	Min	Max
Total facility expenditure (GH₵)	87	94,619.4	99,998.86	12,709.96	613,378.40
Total number of personnel at facility	87	20.29	17.16	6.00	118.00
Total revenue (GH₵)	85	29,833.54	44,130.29	250.00	244,412.50
Total facility beds	87	4.03	3.71	1.00	15.00
Neonatal deaths	47	0.11	0.48	0.00	3.00
Maternal deaths	47	0.06	0.44	0.00	3.00
Outpatient visits	87	3311.81	3917.55	5.00	21,900.00
Years	87	13.06	13.66	1.00	65.00
Outpatient examination rooms	87	1.29	0.69	0.00	4.00

Source: Authors’ computation

All statistics are for the year 2011. Exchange rate in 2011 was US$1.00 = GH₵1.50

On average, there was about 20 personnel in a facility with minimum and maximum values of 6 and 118, respectively. Average annual facility revenue in 2011 was also computed to be GH₵29,833.54 (US$19,889.03) with minimum and maximum values of GH₵250.0 (US$166.67) and GH₵24,4412.5 (US$162,941.67), respectively. Average outpatient visits across facilities was 3311 with minimum and maximum values of 5.00 and 21,900.00.

### Stochastic production frontier

Table 2 shows the estimated production frontier for the facilities included in the analysis. Two different models were estimated. Model 1 included basic inputs while Model 2 included other variables that could indirectly influence productivity. The estimated parameters are generally reasonable for both models. An important component of the production function is to determine the presence of inefficiency among the decision-making units (DMUs). This is observable from the value and significance of lambda (λ). In Table 2, the value of λ is higher (1.70) in Model 2 compared to 1.64 in Model 1. This shows the presence of inefficiency in the estimated production function. The variance, which is decomposed into σ_u_ (inefficiency term) and σ_v_ (normal error term), shows that the inefficiency term dominates the variance. However, efficiency estimates were predicted from Model 2 due to its higher value for lambda.

**Table 2:** Estimated Stochastic Production Frontier

Variables	Model 1	Model 2
Labour	0.40294***	0.38433***
	(0.14137)	(0.14463)
Number of beds	0.59457***	0.47060***
	(0.13958)	(0.14395)
Facility age		0.14165
		(0.11878)
Number of rooms		0.43615
		(0.26747)
Constant	6.68721***	6.47968***
	(0.35257)	(0.35501)
σ_u_	1.02468***	1.01691***
	(0.17372)	(0.16833)
σ_v_	0.62598***	0.59631***
	(0.10402)	(0.09954)
Λ	1.63693***	1.70535***
	(0.23981)	(0.23026)

Source: Authors’ computation

*** significance at 1, 5 and 10% respectively. Standard errors are in parenthesis

### Efficiency estimates

Table 3 shows results of average efficiency estimates across health centers in Ghana. The results indicate an average efficiency estimate of 0.51. This shows an average wastage of about 0.49. This implies that there is room for primary health facilities in Ghana to use fewer resources to produce their current output levels. Tabulating the efficiency scores across various health facility characteristics show some disparity between urban and rural health centers in terms of efficiency performance. Average efficiency among rural facilities was 0.49, relatively lower than urban facilities with efficiency score of 0.51. On average, private facilities recorded significantly higher efficiency score (0.65) than public facilities (0.50). There was also evidence of marked regional disparities with the Northern and Greater Accra regions being the worst efficiency performers. The Brong Ahafo, Ashanti and Central regions were among the best performers. Expectedly, facilities on the performance based financing (PBF) scheme were more efficient than their counterparts off the scheme.

**Table 3:** Mean of efficiency scores by facility characteristics

Variable	Mean	Standard Error
Facility location		
Rural	0.49	0.02
Urban	0.51	0.06
Facility type		
Private	0.65	0.05
Public	0.50	0.02
Regional Location		
Ashanti	0.59	0.05
Brong Ahafo	0.57	0.03
Central	0.65	0.03
Eastern	0.54	0.05
Greater Accra	0.37	0.09
Northern	0.26	0.07
Upper East	0.65	0.05
Upper West	0.49	0.06
Volta	0.46	0.09
Western	0.50	0.06
Performance Based Financing status		
No	0.51	0.03
Yes	0.66	0.06
Mean	0.51	0.22

Source: Authors’ computation

### Potential saving from efficiency

In Table 4, averages of potential savings from improved efficiency were computed and reported across various facility characteristics. The potential savings shows each facility’s deviation from the optimal efficiency level (1.00 in this case). These deviations are then used to compute proportions of revenue that is not realized (or lost) due to such inefficiencies. The estimates show that, on average, health centers could save about GH₵10,593.07 (US$7062.05) per annum. These could serve as additional fiscal space from within the available resource envelope. Based on facility characteristics, the result shows that, relative to their rural counterparts, urban facilities could save higher proportions of their available resource envelope from improved efficiency. Private facilities also have higher fiscal space compared to their public counterparts. Further disparities were identified for various regions and affiliation to the performance based financing scheme in Ghana.

**Table 4:** Average potential savings from efficiency by facility characteristics

Variable	Mean (GH₵)	Standard Error
Facility location		
Rural	7528.61	841.93
Urban	19,647.16	5271.72
Facility Type		
Private	38,395.9	8499.89
Public	9600.114	1484.612
Administrative region		
Ashanti	19,114.26	6556.09
Brong Ahafo	5714.15	4958.34
Central	25,737.83	10,556.45
Eastern	7763.96	1473.81
Greater Accra	6559.77	2754.89
Northern	8591.75	3815.11
Upper East	11,834.81	2530.16
Upper West	8448.45	3159.05
Volta	5007.98	1461.34
Western	5552.22	1910.23
Performance Based Financing		
No	10,088.67	1656.78
Yes	25,312.6	17,148.66
Mean	10,593.07	14,548.3

Source: Authors’ computation

Average exchange rate in 2011 was US$1.00 = GH₵1.50

### Public-private efficiency disparities

Further analysis was conducted to estimate the efficiency differences between public and private facilities. We sought to answer two questions here: (i) what is the extent of the gap between private and public facilities and (ii) what explains the efficiency gap between these facility types? A positive (negative) gap indicates that, on average, public facilities are worse-off (better-off) than private facilities. The estimates show that private facilities were about 44% (Δ = 0.442) more efficient that public facilities (Table 5).

**Table 5:** Decomposition of the public-private PHC facility efficiency gap

	Coefficient
Total Gap (Δ)	0.442
Decomposition of Δ	
Δ_0_	0.704
Δ_pu_	−0.146
Δ_pr_	−0.116
Δ_x_	0.000
% Public in Common Support	0.419
% Private in Common Support	0.667

Source: Authors’ computation

A decomposition of this efficiency gap after matching the facilities on region, age of facility, number of trainings organized for staff, PBF status and location, reveals that −26.2% (Δ_pu_ = −14.6% and Δ_pr_ = −11.6%) is accounted for by differences in support. The common support ensures that analysis is conducted only on facilities with comparable characteristics. The negative value indicates that there are more public facilities that could not be matched to private facilities with comparable characteristics. This part of the gap explains the difference between public facilities that are matched with their private counterparts and those public facilities that remain unmatched. This implies that this gap will be eliminated if all public facilities were matched with private facilities. We also found that the efficiency gap is explained by factors beyond the matching variables (variables not included in this analysis) with Δ_x_ = 0.00% and Δ_0_ = 70.4%.

## Discussion

In general, the findings of the study suggest that there is potential for additional fiscal space to be created through improved efficiency. This supports the theoretical proposition that improved efficiency is an essential source of fiscal space for the health sector [21]. The findings also support some empirical evidence that have showed that significant inefficiencies across health systems in developing countries have increased potential efficiency gains and resource savings [22]. Novignon and Nonvignon [23] found that at the macro level, average potential savings in health expenditure from improved efficiency ranged between 8.1 and 2.2% of Gross Domestic Product. Other authors like Belay and Tandon [24] and Okwero et al. [25] provided evidence to show that improved efficiency in the health sector is as important as increasing resources to the health sector.

The findings of the study also contribute to recent debates about improving primary health care and treating it as the gateway to the health sector. Several authors have argued that a fundamental reform of health systems in several developing countries should include improving service delivery at the primary level [2, 3]. This will also require increasing resource commitments to the lowest levels of the health system. This study provides evidence to show that improving efficiency and hence, saving resource wastages, should be a core part of primary health care reforms.

The foregoing discussions call for relevant policies targeted at ensuring that health sector resources, particularly at the primary level are used efficiently. Efforts in this regard include instituting strong and effective monitoring and evaluation mechanism at various sub-national levels. In Ghana, this may include strengthening district health monitoring and evaluation teams. The recently introduced performance based financing (PBF) scheme for health facilities in the country could be another step in the right direction, if it is effectively implemented. Indeed, the findings of the study show that health facilities that are on the PBF scheme have higher efficiency score relative to facilities not on the scheme. Zeng et al. [26] also provided evidence to confirm the relevance of PBF in health service delivery at the primary level. They found that in Haiti, even modest incentives from PBF schemes were associated with notable growth rates in service delivery.

It is worth noting that gains from efficiency improvement does not increase the resource envelop like increased government allocations or external resources. This implies efficiency gains should not be treated as substitutes for further resource investment but rather complement to efforts by government and stakeholders to increase investment in the sector in order to improve health service delivery.

## Conclusion

The study set out to estimate the level of efficiency across primary health facilities in Ghana. The study also estimated potential efficiency gains and examined disparities in efficiency between primary and public facilities. Data from the ABCE project was used. The SFA and Nopo decomposition procedure were employed in the analysis. The study found evidence of inefficiency across primary health facilities in Ghana. Average efficiency score was estimated to be 0.51 which suggests an average wastage of about 0.49. Average potential resource savings was estimated to be GH₵10,593.07 of total facility revenue. The Nopo decomposition analysis showed there was disparities in efficiency between public and private facilities with an estimated gap of about 44%. Improving monitoring and evaluation mechanisms and employing structured financing schemes such as the PBF scheme may be a step in the right direction.

Some limitations of the study are worth mentioning. First, there was limited data on variables that would have helped to further explore predictors of inefficiency across facilities. For instance, the various expenditure line items in the facilities would have been helpful but not available. Second, the study could have also benefited from some sensitivity analysis to understand how marginal changes in level of efficiency affects the potential gains from the fiscal space created. Such analysis would have provided answers to questions about how much more fiscal space could be generated if a facility improves its efficiency by 1%, which could be addressed by future studies. Finally, the number of facilities and nature of variables included in the study were determined by data availability, which may limit the extent to which the findings of the study can be generalized.

## Additional file

Additional file 1: PHC supplementary file. Estimated Stochastic Production Frontier from Translog specification. This file contains results of the Translog estimation of the stochastic production frontier. The Translog specification is an alternative to the Cobb-Douglas function. Both functions were estimated and the Cobb-Douglas was preferred for this study. (DOCX 13 kb)

## Footnotes

1 Detailed presentation of the analysis is presented in Nopo (2014) and applied in Musa (2014).
