# Supplementary material for: Improving primary health care facility performance in Ghana: efficiency analysis and fiscal space implications
Source: BMC Health Serv Res. 2017 Jun 12;17:399. doi: 10.1186/s12913-017-2347-4 (PMC5468971; doi:10.1186/s12913-017-2347-4)
Supplement: Additional file 1: — PHC supplementary file. Estimated Stochastic Production Frontier from Translog specification. This file contains results of the Translog estimation of the stochastic production frontier. The Translog specification is an alternative to the Cobb-Douglas function. Both functions were estimated and the Cobb-Douglas was preferred for this study. (DOCX 13 kb) [file 12913_2017_2347_MOESM1_ESM.docx]

**Improving primary health care facility performance in Ghana: Efficiency analysis and fiscal space implications**

**Additional file 1**

Appendix 1: Estimated Stochastic Production Frontier from Translog specification

| Variable | Coefficient | Standard error |
| --- | --- | --- |
| Labour | 1.22387 | 0.95786 |
| Number of beds | -0.44529 | 0.71573 |
| Age | 0.79894 | 0.65257 |
| Number of rooms | 1.16599 | 1.72050 |
| Labour square | -0.40110 | 0.39052 |
| Bed square | -0.16857 | 0.37819 |
| Age square | -0.41001 | 0.21802 |
| Rooms square | -0.31703 | 1.98610 |
| Rooms*Age | -0.18400 | 0.65933 |
| Rooms*Bed | -0.75952 | 1.20740 |
| Rooms*Bed | 1.02010 | 1.19752 |
| Labour*Beds | 0.49133 | 0.49469 |
| Age*Beds | 0.29270 | 0.30911 |
| Age*Labour | 0.07763 | 0.40827 |
| Constant | 5.296836** | 1.30508 |
| σ_u_ | 1.0577*** | 0.20455 |
| σ_v_ | 0.55331*** | 0.13643 |
| Λ | 1.91158*** | 0.30886 |
